# Plant Non-Specific Lipid Transfer Proteins (nsLTPs): Comprehensive Functional Analysis and Defense Mechanisms

**DOI:** 10.3390/biology15050417

**Published:** 2026-03-04

**Authors:** Bikram Giri, Dhirendra Kumar

**Affiliations:** 1 Department of Biomedical Sciences, East Tennessee State University, Johnson City, TN 37614, USA; girib1@etsu.edu; 2 Department of Biological Sciences, East Tennessee State University, Johnson City, TN 37614, USA

**Keywords:** nsLTPs, lipid transfer protein, plant defense, abiotic stress, biotic stress, subcellular localization

## Abstract

Non-specific lipid transfer proteins (nsLTPs) play a crucial role in lipid transport across membranes, contributing to cellular integrity and structural stability. These proteins are characterized by the presence of eight conserved cysteine residues that form four disulfide bonds and a hydrophobic cavity that is essential for lipid binding and transport. Interactions of nsLTPs with diverse ligands enable them to participate in key biological processes, including signal transduction, protein folding, membrane stabilization, and cell wall organization. Additionally, these proteins are integral to plant responses to abiotic and biotic stresses and to developmental processes, including growth, germination, and flowering. The interaction between nsLTPs and plant signaling molecules activates regulatory networks that modulate stress-responsive gene expression, reinforcing plant resilience under adverse conditions. Despite their functional significance, the evolutionary trajectory, subcellular localization, and regulatory mechanisms governing nsLTP expression remain limited, as reflected in previous reviews on nsLTPs. This review provides a comprehensive analysis of nsLTP evolution, roles in plant defense and signaling, functional diversity, updated subcellular localization, and future research directions based on recent findings.

## Simple Summary

Non-specific lipid transfer proteins (nsLTPs) play a crucial role in transferring lipids across plant tissues and membranes. They are important for plant defense, particularly under stressful environmental conditions. They achieve this by coordinating signaling pathways and regulating the plant’s antioxidants and hormone systems. nsLTPs are broadly classified into two main groups, nsLTP1 and nsLTP2, based on their molecular size. However, their specific functions remain poorly understood. This paper provides a comprehensive review of nsLTPs, covering their evolution, localization in plants, and biological roles, providing a clear understanding of these important proteins.

## 1. Introduction

Non-specific lipid transfer proteins (nsLTPs) are a unique family of proteins that transfer lipid molecules such as fatty acids, acyl-CoA, phospholipids, and glycolipids [1]. In addition to their basic function in lipid translocation, nsLTPs support important biological processes by linking structural configurations, evolutionary patterns, and subcellular localization. The primary research focus has been on the localization of nsLTPs and their roles in plant growth, development, and defense. Recent advances in gene-editing and breeding techniques across multiple traits and varieties have expanded opportunities to explore how nsLTP gene expression is regulated [2].

Most nsLTPs belong to multigene families. Members of these families exhibit functional diversity, as evidenced by expression across various tissues and at multiple stages of growth and development, and respond to stress [3]. All nsLTPs share several conserved traits, including small molecular size, stable domain structure, and high resistance to denaturing agents. Additionally, the presence of an N-terminal signal peptide facilitates their secretion and subcellular localization [4]. Despite our understanding of nsLTPs in plants, their precise biological roles, structural relationships, and lipid-transfer mechanisms remain poorly studied. Further research is needed to elucidate their specific roles in plant development, stress responses, and metabolism. This review highlights recent advances in nsLTP research and examines their functional significance across diverse biological processes.

## 2. Evolution and Structural Confirmation of nsLTPs

**Figure 1:**
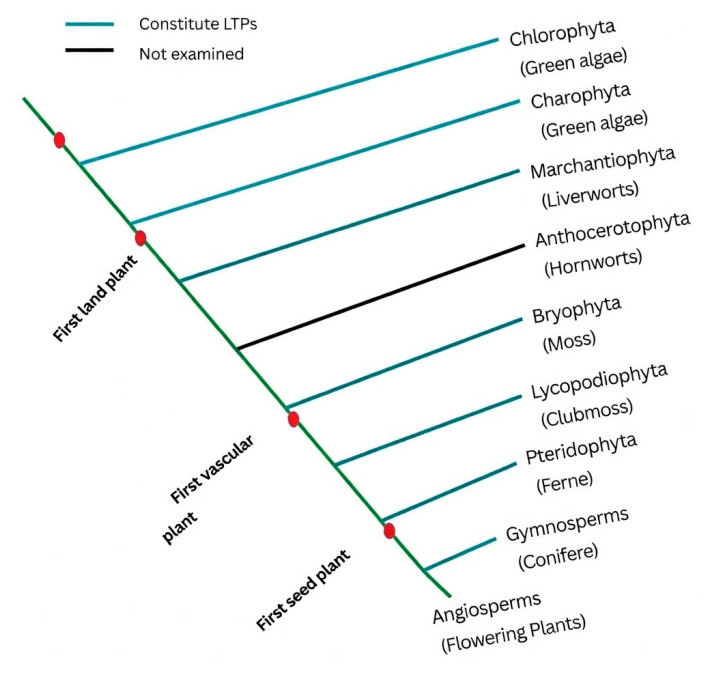
Evolution and occurrence of nsLTPs across different plant species. The line colors indicate the presence of nsLTPs. Blue branches in the cladogram indicate the presence of nsLTPs; black branches indicate no examination yet. Red dots mark vital events in plant evolution. The figure represents modified information [5].

Various reports suggest that the evolution of plant nsLTPs is linked to stress adaptation and defense [5,6]. As described [7], the first lipid transfer protein was isolated from potatoes. Later, spinach leaves were used to purify and characterize nsLTPs, which were named for their ability to mediate in vitro phospholipid transfer between membranes [6]. Recent genome-wide characterization of nsLTP gene families has revealed 79 members in *Arabidopsis*, 156 in wheat, 64 in tomato, 58 in sorghum, 63 in maize, 77 in rice, and 189 across three *Gossypium* species [8,9]. Most nsLTPs isolated and characterized so far belong to flowering and non-flowering plants (Figure 1) [5]. The conservation of *nsLTP* gene families is largely attributable to gene duplication in angiosperms [10]. The nsLTPs are encoded by multi-gene families with more than 40 members in flowering plants and 50 members in bryophytes and ferns. Further, phylogenetic analysis has significantly advanced our understanding of the evolution and functionality of the nsLTP family [8]. However, in non-flowering plants, low sequence homology has challenged the classification of nsLTPs [5].

**Figure 2:**
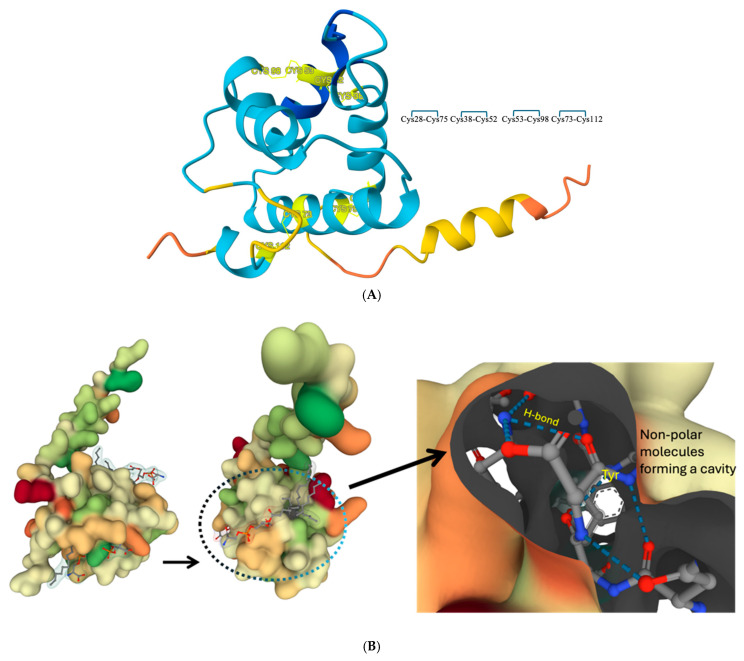
(**A**). 3-D protein model of nsLTP12 from *Arabidopsis thaliana*. The model shows a common structural feature among all nsLTPs: eight cysteine residues. These residues facilitate the formation of four disulfide bonds (connected by yellow lines and showing which cysteine forms the bonds), contributing to the protein’s stability. (**B**). The phosphatidylserine ligand binds within the hydrophobic cavity of the nsLTP12 protein ((**B**), center). The ligand is stabilized by specific molecular interactions, including hydrogen bonding (H-bond) with key residues such as tyrosine (Tyr) ((**B**), far right). Surrounding nonpolar residues contribute to forming a hydrophobic pocket that facilitates ligand accommodation and stability within the binding site.

Despite variations in amino acid sequence, identities among nsLTPs range from 50 to 90% across plant species. Most nsLTPs share common characteristics, including small protein size (6–15 kDa) and presence of an eight-cysteine motif (8CM) arranged as C-Xn-C-Xn-CC-Xn-CXC-Xn-C-Xn-C, where C represents ‘Cysteine’ and ‘Xn’ is an amino acid variable number (Figure 2A) [11]. They possess an N-terminal signal peptide that directs the protein to specific subcellular locations, and a hydrophobic tunnel-like cavity, formed by four disulfide bridges, facilitates the lipid transfer (Figure 2B) [5,12].

Although computational modeling has been instrumental, the pivotal advances in understanding the structural biology of nsLTPs have stemmed from experimental structural elucidation. As of January 2026, the Protein Data Bank (PDB) contains 25 nsLTP structures, determined by techniques such as X-ray diffraction and Nuclear Magnetic Resonance (NMR) spectroscopy. A distinct characteristic of nsLTPs is a hydrophobic tunnel, noted for its adaptability, which accommodates single- and double-chain lipids [12]. In most cases, following ligand binding, such as phosphatidylglycerol and 1,2-dimyristoyl phosphatidylglycerol, the helix position of the N-terminal and C-terminal in 8CM expands the hydrophobic cavity by two to four times, reflecting the intrinsic mechanism of lipid binding and transfers [11,13]. The binding of lipid molecules occurs through hydrophobic interactions within the cavity. Primarily, non-polar residues, such as leucine, isoleucine, phenylalanine, and valine, engage with lipid tails. The ligand binding of phosphatidylcholine to LTP12 is shown in Figure 2B, where a hydrogen bond forms between the ligand carboxyl group and the hydroxyl group of tyrosine. The confirmational flexibility studied using molecular dynamics (MD) simulations and crystallographic studies highlights cavity expansion and binding of fatty acids like oleic acid and myristic acid [13]. This observation is further supported by studies involving maize (*ZmLTP1.6*), rice (*OsLTP1.18*), and tobacco (*NtLTP1.1*) *nsLTPs*, where the cavity orientation changes upon binding of the ligand to the protein driven by differences in sequence loop regions on H1-H2 [14,15,16,17].

The exchange of lipids occurs between organelles where nsLTPs are found at membrane contact sites (MCS). Transport simulations suggest a non-vesicular shuttle mechanism, where nsLTPs do not require metabolic energy to transfer lipids as lipid contact hydrophobic cavity rather than aqueous environments, which are less favorable [18,19]. Although some of the lipids are transported against a concentration gradient requiring energy, as in ATP-binding cassette transporters [20,21,22,23]. While information on in vivo transport lacks direct observation, there has been in vitro evidence suggesting involvement of MCS in efficient lipid trafficking [24]. MCS also shortens the diffusion distance for nsLTPs, accelerating lipid transfer [25]. As discussed earlier, disulfide bonds stabilize the nsLTPs to maintain a rigid extracellular alpha-helical structure for stable binding of hydrophobic lipid signals, such as oxylipins, within the internal cavity. The MCS helps in transporting lipid cargo at the plasma membrane by leucine-rich repeat receptor-like kinases (LRR-RLKs). The ligand recognition complexes promote the receptor conformational change, triggering kinase activation and downstream signaling, involving activation of mitogen-activated protein kinase (MAPK) cascade and the activation of systemic acquired resistance (SAR) [26,27]. Nevertheless, whether nsLTPs act as lipid shuttles or multifunctional scaffolds for signaling remains unclear, and resolving this requires integrating studies using in vivo imaging, functional assays, and advanced structural dynamics (EM/NMR/MD simulations).

## 3. Classification of Plant nsLTPs

In plants, nsLTPs are small, basic proteins and account for up to 4% of total soluble proteins [7]. Initial classification of nsLTPs was based on molecular mass. The nsLTP1 (Type I) family members were around 10 kDa, and the nsLTP2 (Type 2) were 7 kDa proteins [14]. The classification of nsLTPs was regularly updated based on structural features, sequence similarity, hydrophobic cavities, and other broad classifications [4,8,28,29,30,31,32]. Boutrot has developed a classification based on sequence similarity and cysteine residue spacing, with roles in SAR supported by antimicrobial and stress regulatory motifs. Based on this, the nsLTPs from rice (*Oryza sativa* L.), *Arabidopsis thaliana*, and wheat (*Triticum aestivum* L.) were classified into nine distinct types (Types I–IX) (Table 1) [28]. This was later expanded to include Types X and XI. Type X nsLTPs represented a new group that included nsLTPs from *Solanaceae* [31].

The classification was further refined by Edstam et al. [5], incorporating the presence of the GPI anchor (Types 1, 2, C-K). Subsequently, Wang et al. [4] proposed a revised system based on the pattern of the 8CM and categorized the nsLTPs into Types I–V. More recently, Fleury et al. [8] combined sequence alignment, phylogeny, and structural biochemistry to classify nsLTPs into two types, Type I and Type II, as suggested by Boutrot et al. [28]. A 2023 study by Huang et al. [33] described a new class of nsLTP from algae distinguished by an extended 8CM spacing, larger molecular mass (10.36–50.28 kDa), and lower pI (<8). Further analysis is needed to determine if this new group of nsLTPs falls into either the updated group of Type I or II described by Fleury et al. [8].

Compared to early classification model systems, the newer approaches are based on the spacing of eight cysteine motifs, the presence of glycosylphosphatidylinositol (GPI) anchors, and the use of advanced bioinformatics tools such as AlphaFold, molecular docking, and PDB’s pairwise structural alignment. Using the computational approach will help to characterize novel subfamilies of nsLTPs and understand diverse roles in defense and plant physiology. The nsLTP classification systems are summarized in Table 1.

## 4. Spatial Expression and Cellular Localization of Plant nsLTPs

**Figure 3:**
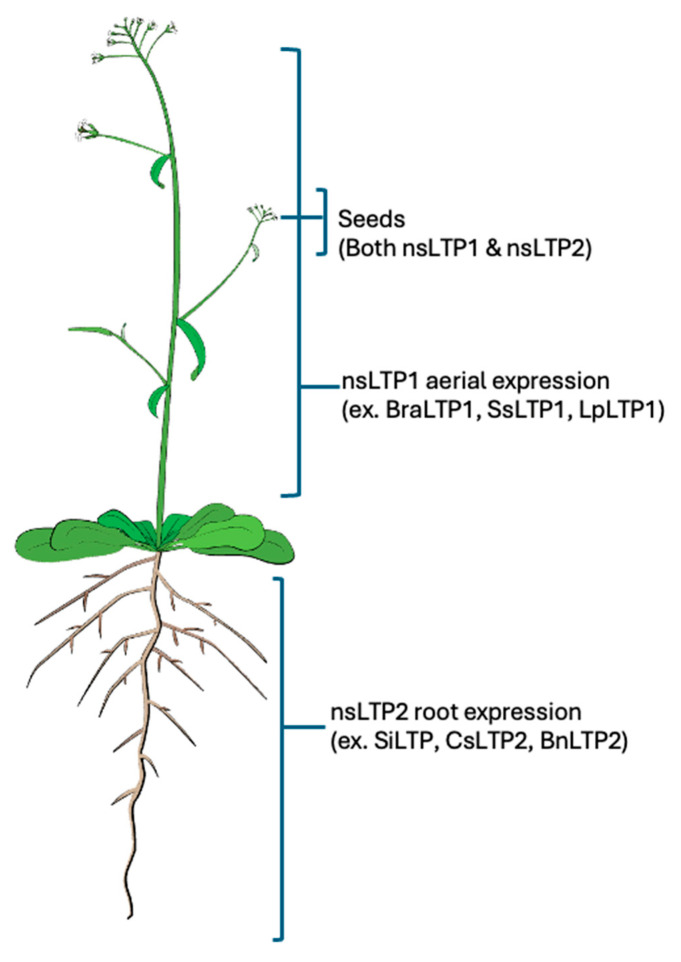
Localization of nsLTPs. Tissue-specific expression of nsLTPs. nsLTP1s are most expressed in the aerial parts, while nsLTP2s are expressed in roots. However, seeds express both nsLTP1s and nsLTP2s.

The cellular expression of nsLTPs varies among plant species, providing diverse biological functions. Different plant organs are known to express nsLTPs, including seeds, leaves, stems, roots, flowers, and fruits [21]. The expression profile and localization differ among nsLTP types, with nsLTP1s present in cuticle-covered epidermal cells and embryonic and vascular tissues [22]. The most widely recognized and abundant type, nsLTP1, is found in the aerial organs of plants, while nsLTP2s are expressed in roots (Figure 3). Seeds contain both nsLTP types [23]. In seeds, diverse physiological functions are supported by multiple localizations within the cell wall, plasma membrane, and extracellular space [24].

Data from diverse species have provided crucial information on the localization of nsLTPs and their role in plants. The extracellular localization of some nsLTPs has been reported, including in barley [25], carrot [26], *Arabidopsis* [27], tobacco [28], and soybeans, having a potential role in forming the cuticle barrier and antimicrobial property [29]. Additionally, research has challenged the apoplastic protein tag, revealing that nsLTPs are expressed at the plasma membrane, exhibit intracellular localization, and associate with the intracellular matrix, providing intracellular lipid transport and defense signaling. The barley, *Arabidopsis*, grapevine, tobacco, and rice nsLTPs are localized outside the cell [30]. While some nsLTPs from castor bean seeds (*RcLTP*) are intracellularly localized (glyoxysomes), facilitating intracellular lipid mobilization, the cowpea seed nsLTP (*VuLTP*) is in vacuoles, having a role in lipid homeostasis, and the pepper seed nsLTP (*CaLTP1*) is in vesicles and the cell wall involved in lipid trafficking [31].

In some cases, nsLTP localization is dynamic. Based on cell shape and cell wall curvature during cell growth and differentiation, the *A. thaliana AtLTPg* localizes to the apoplast, plasma membrane, and cell wall, assisting in cuticular wax deposition and cell wall fortification [34]. Similarly, *HaAP10*, an nsLTP from *Helianthus annuus* seeds, localizes to the apoplast and plasma membrane in dry seeds and upon imbibition, relocates to the intracellular matrix, highlighting the versatility of nsLTPs in adapting lipids and regulating subcellular trafficking [35]. Additionally, the cellular localization of type G nsLTPs (*LTPGs*) is regulated by alternative splicing, resulting in two distinct transcripts. One contains the GPI anchor signal for cuticle integrity and lipid deposition, whereas the non-anchored isoform potentially supports antimicrobial defense and lipid shuttling. Splicing events enable differential localization of LTPGs in response to varying tissue types and environmental conditions [5,36]. Various approaches and methods have been used to investigate the subcellular localization of nsLTPs, as detailed in Table 2. Proteins identified as extracellularly localized were obtained from cell culture or from proteomic analysis of apoplastic fluid. Similarly, immunochemical studies highlighted the association of nsLTPs with cell wall locations. Furthermore, localization varies with developmental stage and environmental conditions. Therefore, a combination of experimental methods under different conditions is required to enhance the robustness of detecting the subcellular localization of nsLTPs [37]. Furthermore, future studies could use mass spectrometry-based lipidomics to understand organelle trafficking of lipids during stress, providing clues on their mobilization and interaction with nsLTPs.

## 5. Biological Significances of nsLTPs

**Figure 4:**
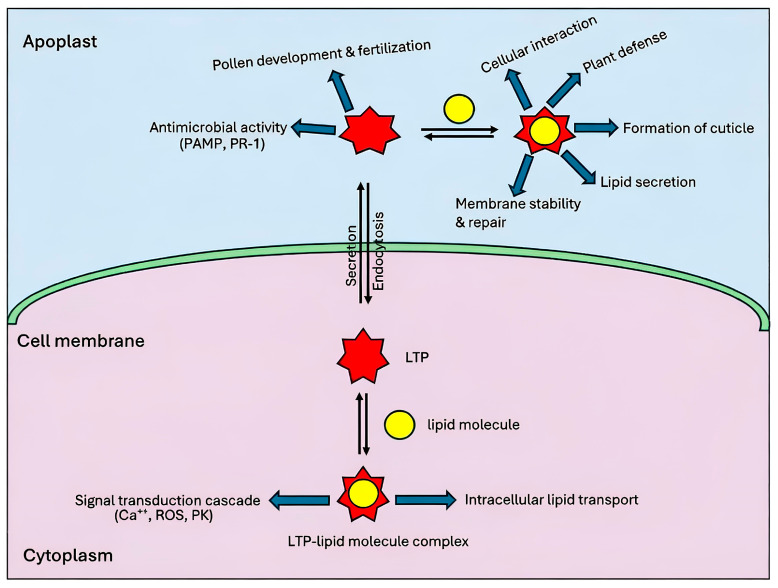
Schematic representation of the potential function of LTPs in plants. Distinct roles, such as activation of the signal cascade and intracellular lipid transport in the cytoplasm. The interaction in the apoplast functions for plant defense, cuticle formation, secretion of lipids, and pathogenesis-related protein activation.

In recent years, extensive research has elucidated the diverse functions of nsLTPs, revealing their involvement in numerous physiological responses (Figure 4). These proteins are implicated in growth and reproduction, defense against pathogenic microbes, symbiosis, stress adaptation, and antimicrobial activity [55]. However, the precise mechanisms by which nsLTPs operate across these processes remain poorly understood.

### 5.1. Defensive Functions of nsLTPs Under Biotic Stress

Numerous studies have reported the expression of nsLTPs in response to attacks by bacterial and fungal pathogens, such as wheat, potato, and rice [56,57,58]. nsLTPs exhibit antimicrobial and antifungal properties, inhibiting the growth of pathogenic bacteria and fungi, including *Pseudomonas solanacearum*, *P. syringae*, *Fusarium solani*, *Alternaria brassicola*, and *Botrytis cinerea* [59,60]. The antimicrobial activity of nsLTPs is effective against a specific range of microorganisms. For instance, nsLTPs from Arabidopsis, radish, and onion have demonstrated antimicrobial activity at micromolar concentrations. McLaughlin et al. reported that overexpressing *AtLTP4.4* in transgenic wheat significantly reduced the growth of the pathogen *Fusarium graminearum*, a pathogen that causes diseases in small grain cereals [59]. Additionally, Ace-AMP1 from transgenic wheat exhibits an antifungal response to *Blumeria graminis* [61], while rice and onion have demonstrated both fungal and pathogen resistance [62]. Further findings revealed that Durum wheat nsLTP1 (*TdLTP4*) exhibits anti-inflammatory, antimicrobial, and antifungal properties and plays a crucial role against human and foodborne pathogenic bacteria [63]. Interestingly, nsLTPs also synergize with other antimicrobial peptides, such as defensins and thionins, forming a broader network of immunological proteins in plant physiology [64]. Furthermore, *BrLTP2.1* from *Brassica rapa* found in nectar displayed both antifungal and antimicrobial activities [65].

Plant immunity often involves recognition of pathogen attacks via pathogen-associated molecular pattern (PAMP) signaling, triggered by bacteria and fungi, which in turn elicits a defense response mediated by hydrophobic ligands (Figure 4) [66]. Additionally, nsLTPs in plants are categorized as pathogenesis-related proteins within the PR14 family, which are specialized for plant defense [36]. Specifically, type IV nsLTPs in Arabidopsis form complexes with glycerol-3-phosphate, facilitating their translocation and signaling induced SAR, providing broad-spectrum protection against pathogens [67]. This function highlights the essential role of nsLTPs in plant immune response pathways, contributing to effective resistance to pathogenic attacks. Similarly, overexpression of nsLTP genes such as *CaLTP* and *CaLTP2* in pepper, *LjAMP1* and *LjAMP2* in motherwort, and nsLTP in barley has shown significant resistance to bacterial and fungal pathogens [68,69].

Furthermore, signaling molecules such as abscisic acid, salicylic acid, ethylene, and methyl jasmonate trigger a signaling cascade that regulates nsLTP gene expression. Recent studies have identified the involvement of jasmonates with various saturated and unsaturated fatty acids in *LcLTP2* (*Lens culinaris*) and *BrLTP2* (*Brassica rapa*) to stimulate defense response [65]. Interestingly, research on wheat *LTP1* has shown that it interacts with elicitin, an elicitor protein from oomycetes, potentially competing for binding sites on tobacco cells for plant defense [64]. The interaction, enhanced by the presence of jasmonic acid (JA), has been linked to increased resistance against the pathogen *Phytophthora parasitica* [70]. Nonetheless, many such findings are based on in vitro experiments and may behave differently in vivo.

### 5.2. Defensive Functions of nsLTPs Under Abiotic Stress

nsLTPs enable plants to adapt to diverse environmental conditions, such as high salinity [71,72], drought [45,73], and freezing stress [43,74]. In bread wheat (*Triticum aestivum*), *TaLTP3* is induced by cold, drought, ABA, and oxidative stress [75]. The transcription factor MYB96 regulates the expression of various *nsLTP* genes, including *LTP3*, coordinating plant responses to freezing and drought stress in *Arabidopsis* [45]. Stress-responsive motifs (TGA, TCA, and ABRE) and signaling cascades with ROS homeostasis in *HcnsLTP111* are reported to regulate drought and stress tolerance in Kenaf (*Hibiscus cannabinus*) [76]. Further, overexpression of nsLTPs, such as *OsDIL* in rice, *LTP3* and *AZI1* in *Arabidopsis*, and *CaLTP1* in pepper, significantly enhances tolerance to abiotic stress.

Across multiple monocot and dicot species, such as rice, wheat (*TaLTP40*, *TaLTP75*, *TdLTP4*, *TdLTP2*), *Arabidopsis* (*AtGBF3*), cabbage, tobacco (*NtLTP4*), foxtail millet (*SiLTP*), nsLTP overexpression has been shown to enhance tolerance to various abiotic stresses [77,78,79,80,81]. The cabbage-derived nsLTP WAX9 also provides cryoprotection to isolated spinach thylakoids [82]. In addition, plants overexpressing nsLTPs altered sensitivity to key stress-related hormones, including SA, JA, and ABA [64,83]. Recent findings by Zhou et al. showed that three members of wheat nsLTP, located on chromosomes 1B, 5D, and 7B, enhance protein abundance and mediate salt tolerance in the wheat overexpression line *TaNRX1-2D* [84].

### 5.3. Role of nsLTPs in Cuticular Wax Deposition

Long-chain fatty acids (C20–34) and their derivatives make up waxes, whereas a polymer network of C16 and C18 fatty acids makes up plant cutin layers [85]. Insulation, stomatal water loss prevention, radiation damage reduction, and defense against pathogen attacks, including herbivores, are all provided by these structures. Notably, lipid metabolism in epidermal cells primarily targets cuticular lipids [86]. Plant colonization success relies on molecular barriers formed by lipid polyesters [12]. Research has shown that cuticular and wax polymer synthesis occurs in the apoplast, potentially facilitated by nsLTPs trafficking monomers [87,88]. Interestingly, nsLTP expression is notably higher in young, developing tissues synthesizing surface wax in species such as *Brassica oleracea* and *Arabidopsis* [39]. Within the extracellular matrix, cell walls, or plasma membrane of epidermal cells and secretory tissues, nsLTPs contribute to cutin monomer deposition [89,90]. LTPGs, which contain GPI anchors, are implicated in the biosynthesis and accumulation of suberin and cutin monomers, and disruption of GPI-anchored gene *LTPG1* in *Arabidopsis* resulted in altered cuticular lipid composition, increasing susceptibility to fungal infection [47,91].

Furthermore, nsLTP expression increases in response to environmental stressors, including drought, heavy metal exposure, and heat stress, and correlates with wax deposition [92,93]. Under stress conditions, broad classes of nsLTP1 and nsLTP2 enhance cuticular layer deposition and thickening, preventing pathogen attack with antimicrobial properties [94]. In *Solanum lycopersicum* (*Sltpg3*), nsLTPs delay water loss by thickening the cuticle, reducing permeability, and softening tomato fruits for a longer shelf life [95]. Another study suggests that *Brassica napus BraLTP1* plays a role in enriching epicuticular wax deposition [96]. The *Arabidopsis AtLTP2* and *AtLTP4* were found to link nsLTPs to cuticular wax deposition and to control water permeability [48]. Conversely, knocking out nsLTP expression alters the cuticle’s lipid composition and the density of the cuticle layer [89,97]. Despite these insights, the precise mechanisms by which nsLTPs transport lipid components to form the cuticle remain unclear.

### 5.4. Involvement of nsLTPs in Seed Development

Expression of nsLTPs increases significantly during seed germination and development. In rice, *OsLTPL23* and *OsLTPL18* are key regulators of seed germination [98]. In *Coffea arabica* and *C. canephora*, nsLTP expression is observed in the endosperm and embryo during seed germination [99]. The biochemical and physiological functions of nsLTPs in seed germination include mobilizing stored lipids through the regulation of fatty acid beta-oxidation [42]. Sesame LTP, *SiLTP1.23*, and *SiLTP1.28* (*Sesamum indicum*) play a crucial role in oil accumulation in seeds [9]. Additionally, research from [100] showed that *ElLTP1* and *ElLTP2* in *Euphorbia lagascae* act as protease inhibitors, protecting cotyledons from proteolysis during programmed cell death. In sunflower (*Helianthus annuus*), *HaAP10* relocates to glyoxysomes, the organelles involved in lipid metabolism, after seed imbibition [35]. Five lipid transfer and storage proteins were expressed (ACP, ACBP, DIR1, FPKM), regulating the wax ester synthesis pathway for seed development in Jojoba [101]. Furthermore, nsLTPs are involved in pollen development, as demonstrated by recent studies in chili pepper, *Arabidopsis*, maize, wheat, and rice [44,102,103]. However, the precise pathways and mechanisms through which nsLTPs mobilize lipids during seed development remain not fully understood.

### 5.5. nsLTPs as Modulators of Plant Signal Transduction

**Figure 5:**
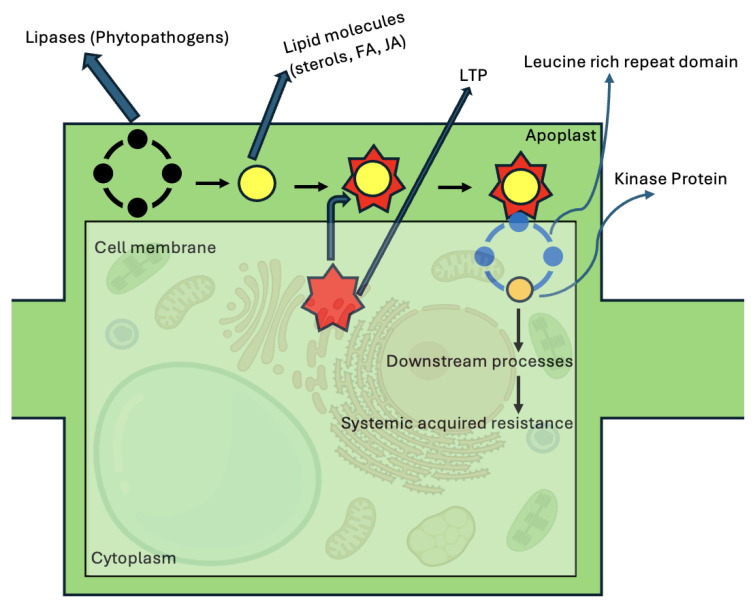
Schematic representation of nsLTP’s role in defense and systemic acquired resistance in a plant cell. The lipases released by phytopathogens convert fatty acids to JA bound by apoplast LTPs, forming LTP-JA complexes that activate LRR receptors, triggering downstream signaling and SAR.

By forming complexes with lipid molecules, nsLTPs play a crucial role in initiating and regulating a variety of signaling pathways in plants. After binding to nsLTPs, these lipid molecules interact with receptors such as serine/threonine protein kinases, which have a transmembrane region, extracellular leucine-rich repeat (LRR) domains, a transmembrane region, and the kinase (PK) domain towards the cytosol. This interaction initiates a signal transduction pathway with adaptable second messengers. Transcription factors, protective agents, pathogenesis-related (PR) proteins, and other antimicrobial peptides (AMPs) are stimulated by the activation of the MAPK cascade [104]. SAR is induced by this chain of events (Figure 5) [30].

One signaling molecule, the oxylipin, is produced from unsaturated fatty acids in response to reactive oxygen species (ROS) and plays a crucial role in enhancing plant growth and development under stress conditions [105,106]. ROS scavenging activity protects plants from oxidative stresses induced by pathogen infections. nsLTPs are directly or indirectly involved in ROS scavenging as shown by studies involving *NtLTP1.38*, *NtLTP2*, *NtLTP25*, and the overexpression of Type I nsLTP in *Nicotiana benthamiana* (*NbLTP1*), conferring resistance to tobacco mosaic virus (TMV) [77,107,108,109,110]. In barley, the combined action of LTPs and oxylipins is known to protect plant cells under stress by neutralizing toxic components [111]. Similarly, in *Cucumis sativus*, *CsnsLTP6* overexpression confers resistance against *Corynespora cassiicola* [112]. This is achieved first through activation of defence signaling pathways and then by rapidly activating the ROS-scavenging enzymes. The LTP2 (*DIR-1*) in *Arabidopsis* and glycerol-3-phosphate (G3P) are involved in SAR development and long-distance signaling [113]. These nsLTPs bind to lipid molecules, for example, oxylipins, fatty acids, produced by lipases during pathogen infection and trigger a signaling cascade that leads to the SAR response (Figure 5) [114,115]. Furthermore, the xylogen culture of *Zinnia*, which has a GPI anchor, binds to plant sterols and is characteristic of Type II nsLTPs [116]. This protein participates in signaling pathways and intercellular interactions. It is suggested that similar proteins in other plants, which have nsLTP domains resembling those of nsLTP2s, may also be involved in intercellular interactions and signal transduction, functioning within a complex that includes a lipid molecule [116]. The MAPK cascade is involved in response to pathogens mediated by nsLTPs, such as *StLTP10* [117], and in *Arabidopsis*, where both camalexin and antimicrobial phytoalexin production are triggered by MAK3/MPK6 signaling [72,118]. As described earlier, nsLTPs contribute to the lipid exchange between organelles at MCS, highlighting the dynamic shuttling of lipids to coordinate cellular resilience and signal immune activation.

## 6. Precise Gene Editing Technology: Future Directions

Despite our understanding of the types and subtypes of nsLTPs, the precise role of nsLTPs under different stress conditions remains poorly elucidated. Researchers still face challenges posed by the absence of information on tissue-specific expression, structural differences in nsLTPs, and the functional diversity of nsLTPs during development. Finally, advances in precise gene-editing technologies have started to circumvent these limitations [119]. The use of CRISPR/Cas9 has changed the traditional approach to plant editing, offering greater precision and efficiency [120]. CRISPR/Cas9 can be used to multistack gene knockout of the nsLTP family to understand their precise role. For instance, gain- and loss-of-function mutants in sesame (*SiLTP1*) have provided critical insights into seed oil accumulation [121], while CRISPR-mediated lysine malonylation in *Dendranthema grandiflorum DgnsLTP1* resulted in resistance to cold [122]. Furthermore, the targeted modification of nsLTP genes through CRISPR could enhance plant resilience to biotic and abiotic stresses [75,112,123].

## 7. Conclusions

In summary, research on nsLTPs has increased over the years; however, their specific functions and localization-based roles remain incompletely characterized. Studies using overexpression and knockout lines have demonstrated the role of nsLTPs in mediating stress responses, highlighting their involvement in lipid binding and transfer, and orchestrating complex signaling networks. As we continue to decode the nsLTPs, advanced genetic engineering techniques and computational modeling are likely to provide an improved approach for the functional characterization of different nsLTPs across plant species.

## Figures and Tables

**Table 1:** nsLTP classification. The examples of nsLTPs identified from distinct species were categorized based on several classification standards. The nsLTP types are based on characteristics such as molecular weight, 8CM spacing, GPI, signal peptide, and phylogenetic cluster. The new group of nsLTP from Algae has been added to the list.

nsLTP Type	Reference	Classification Standard	Signal Peptide	Subcellular Localization	Species	Examples
I, II	[7]	Molecular weight	Yes	Secretory pathway (SP)	Most Monocot/Dicot	*AcvLTP1.1-3*, *AtLTP12*, *PtLTP2.1*
I to IX	[28]	Sequence similarity, intervals of eight cysteine residues	Yes	SP	Rice, *Arabidopsis*, and wheat	*OsLTP1*, *AtLTP2*, *TaLTP5*
I–V & X	[4]	Sequence identity and eight cysteine residue intervals	Yes	SP	*Solanaceae*	*CaLTP2*, *SlLTP1*, *NtLTP3*
I, II, C, D, E, F, G, H, J, K	[5]	GPI modification site, sequence similarity, and cysteine residue spacing	Yes	SP, Mitochondria	Green and red algae, moss, ferns, and conifers	*PtLTPc1-2*, *MpLTPd1-8*, *SmLTPe1-3*, *PtLTPf1*, *MpLTPg1-4*, *SmLTPh1-6*, *PpLTPj1-7*, *PpLTPk1-2*
III, VIII, V, VI, IX, XI, I, II, IV	[8]	Amino acid length (60–150 aa), signal peptide, monodomain	Yes	SP	Wheat, Rice	*TdLTP*, *OsLTP2*
New algal lineage	[33]	Presence of 8CM, signal peptide and phylogenetic cluster	Yes	SP	Algae	*CrLTP1*, *CrLTP2*

**Table 2:** Subcellular localization of nsLTPs. The table summarizes nsLTPs identified across multiple plant species, indicating their subcellular localization, experimental methods used for localization analysis, and associated biological functions, highlighting their functional diversity.

nsLTP	Species	Localization	Method	Functions	Reference
*DIR1*	*Arabidopsis* (*A*. *thaliana*)	Extracellular	Petiole exudate	Systemic Acquired Resistance (SAR)	[38]
*LTP1*	*Arabidopsis* (*A*. *thaliana*)	Cell wall	Immunochemical study	Cuticle assembly	[39]
*PAPI*	Barley (*Hordeum vulgare*)	Extracellular	Cell culture	Defense barrier	[40]
*HaAP10*	Sunflower (*Helianthus annuus*)	Apoplastic, plasma membrane, intracellular	Fluor-immunolocalization studies	Antifungal	[41]
*nsLTP*	Castor bean (*Ricinus communis*)	Glyoxysome matrix and cell wall	Immunolocalization, cell fractionation analysis	Lipid storage	[42]
*SsLTP1*	Eggplant (*Solanum sogarandinum*)	Intracellular	Western analysis	Lipid trafficking	[43]
*LTP12*	*Arabidopsis* (*A*. *thaliana*)	Plasma membrane	Fluor-immunolocalization study	Membrane stabilization	[44]
*LTP3*	*Arabidopsis* (*A*. *thaliana*)	Cytoplasm	*Arabidopsis* protoplast transformation	Lipid binding	[45]
*LTPG*	*Arabidopsis* (*A*. *thaliana*)	Plasma membrane	Transgenic experiment Promoter::YFP-LTPG	Cuticular lipid export	[46]
*PpLTPG2*	*Physcomitrella* (*Physcomitrella patens*)	Plasma membrane	35S::YFP-PpLTPG2 fusion	Lipid export	[47]
*Ca-LTP1*	Pepper (*Capsicum annuum*)	Intracellular vesicles, extracellular space	Immunolocalization, Western blotting	Defense	
*LTP2*	*Arabidopsis* (*A. thaliana*)	Extracellular	TAIR	Wax formation	[48]
*VuLTP*	Cowpea (*Vigna unguiculata*)	Extracellular, cell wall, vacuoles	Immunolocalization of tissue sections	Antimicrobial	[49]
*WAX9*	Broccoli (*Brassica oleracea*)	Cell wall	Immunogold labelling	Wax deposition	[50]
*EP2*	Carrot (*Daucus carota*)	Extracellular	Embryogenic cell culture	Plant development	[51]
*Two LTPs*	Tobacco (*Nicotiana tabacum*)	Extracellular	Proteomic analysis	Defense	[52]
*nsLTP1e1*	Wheat (*Triticum aestivum*)	Aleurone granules	Immunolocalization study	Seed storage	[53]
*Four LTPs*	Grape (*Vitis vinifera*)	Extracellular	Somatic embryo cultures	Embryo lipid barrier	[54]
*AtLTP3*	*Arabidopsis (A*. *thaliana*)	Plasma membrane	Reporter-based transcriptional fusion/plasmolysis	Lipid dynamics	[44]
*AtLTP4*	*Arabidopsis* (*A*. *thaliana*)	Plasma membrane	Reporter-based transcriptional fusion/plasmolysis	Membrane stabilization	[44]
*AtLTP5*	*Arabidopsis* (*A*. *thaliana*)	Cell wall	Reporter-based transcriptional fusion/plasmolysis	Cell wall lipid reinforcement	[44]
*AtLTP6*	*Arabidopsis* (*A*. *thaliana*)	Cell wall	Reporter-based transcriptional fusion/Plasmolysis	Structural lipid support	[44]

## Data Availability

No new data were created or analyzed in this study. Data sharing is not applicable to this article.
